# Serum Inflammatory Factor Profiles in the Pathogenesis of High-Altitude Polycythemia and Mechanisms of Acclimation to High Altitudes

**DOI:** 10.1155/2021/8844438

**Published:** 2021-08-25

**Authors:** Hai Yi, Qianjin Yu, Dongfeng Zeng, Zhaohua Shen, Jiali Li, Lidan Zhu, Xi Zhang, Quanhong Xu, Hu Song, Peiyan Kong

**Affiliations:** ^1^Department of Hematology, Xinqiao Hospital, Army Medical University, PLA, Chongqing 400037, China; ^2^Department of Hematology, The General Hospital of Western Theater Command, PLA, Chengdu 610083, China; ^3^Department of Hematology, Linyi People's Hospital, Dezhou 251500, China; ^4^Department of Hematology, Daping Hospital, Army Medical University, PLA, Chongqing 400042, China; ^5^Department of Gastroenterology, Third Xiangya Hospital, Central South University, Changsha 410013, China; ^6^Department of Health Medicine, No. 956 Hospital of the People's Liberation Army, Nyingchi Prefecture, Tibet Autonomous Region, 860000, China; ^7^Department of Respiratory Medicine, No. 954 Hospital of the People's Liberation Army, Shannan Prefecture, Tibet Autonomous Region, 856100, China

## Abstract

High-altitude polycythemia (HAPC) is a common aspect of chronic mountain sickness (CMS) caused by hypoxia and is the main cause of other symptoms associated with CMS. However, its pathogenesis and the mechanisms of high-altitude acclimation have not been fully elucidated. Exposure to high altitude is associated with elevated inflammatory mediators. In this study, the subjects were recruited and placed into a plain control (PC) group, plateau control (PUC) group, early HAPC (eHAPC) group, or a confirmed HAPC (cHAPC) group. Serum samples were collected, and inflammatory factors were measured by a novel antibody array methodology. The serum levels of interleukin-2 (IL-2), interleukin-3 (IL-3), and macrophage chemoattractant protein-1 (MCP-1) in the eHAPC group and the levels of interleukin-1 beta (IL-1 beta), IL-2, IL-3, tumor necrosis factor-alpha (TNF-alpha), MCP-1, and interleukin-16 (IL-16) in the cHAPC group were higher than those in the PUC group. More interestingly, the expression of IL-1 beta, IL-2, IL-3, TNF-alpha, MCP-1, and IL-16 in the PUC group showed a remarkable lower value than that in the PC group. These results suggest that these six factors might be involved in the pathogenesis of HAPC as well as acclimation to high altitudes. Altered inflammatory factors might be new biomarkers for HAPC and for high-altitude acclimation.

## 1. Introduction

HAPC occurs in 5% to 18% of the population residing on the Qinghai-Tibetan Plateau. It is caused by hypobaric hypoxia and is characterized by excessive erythrocytosis [1]. Excessive erythrocytosis can result in significantly increased blood viscosity, microcirculation disturbances, or even extensive organ damage and a reduction in blood flow velocity, which causes other complications associated with chronic mountain sickness, such as high-altitude pulmonary hypertension and high-altitude heart disease [2]. According to the Qinghai diagnostic criteria in 2004, HAPC is defined as hemoglobin concentration higher than 210 g/L in male and 190 g/L in female, accompanied by the symptoms of breathlessness, palpitations, sleep disturbance, and headache. [1] However, there is little prevention or treatment for this disease because the molecular mechanisms underlying the pathogenesis of HAPC are not well understood. On the other hand, some people can show acclimations to hypoxic environments, which indicated as lower hemoglobin levels, higher oxygen saturation of the blood, higher work performance, and few symptoms than HAPC when residing at high altitude. Previous studies have showed that there might be a genetic basis to the adaptation to high altitude [3–6]. However, other mechanisms which contribute to high-altitude acclimation are still largely unknown and need to be clarified.

It has been reported that upon exposure to high altitude, oxidative stress can occur [7, 8]. Oxidative stress could induce hypoxia-inducible factor 1 (HIF-1) expression so that increase EPO secretion, which leads to erythrocytosis. However, other factors are associated with excessive erythrocytosis and trigger a series of uncomfortable symptoms [9, 10]. Furthermore, inflammatory factors are released in response to hypoxia and oxidative stress [11, 12]. It has been demonstrated that exposure to high altitude is associated with elevated inflammatory mediators [13]. Hypoxia can elicit tissue inflammation, and inflamed tissues consequently become hypoxic. In plateau hypoxia, mononuclear macrophages induce an increase in the levels of inflammatory factors interleukin-1 (IL-1), interleukin-6 (IL-6), and interleukin-8 (IL-8) [14]. This might accelerate the proliferation of bone marrow hematopoietic stem cells and promote excessive erythrocytosis [15, 16]. A recent study shows that IL-6 increases with altitude in children populations. And IL-6 might also increase the level of hepcidin, which may reduce iron availability for erythropoiesis [17]. The relationship between inflammatory factors and erythropoiesis needs to be clarified.

The inflammatory factor profiles in the pathogenesis of HAPC and mechanisms of acclimation to high altitude are largely unknown. Our hypothesis was that some inflammatory factors might contribute to the pathogenesis of HAPC as well as to acclimations to high altitudes. We divided the individuals into four groups: the plain control (PC) group, plateau control (PUC) group, early HAPC (eHAPC) group, and confirmed HAPC (cHAPC) group. The PUC group indicated that the individual in this group were acclimated well in high-altitude areas. In this study, we used a novel technique to simultaneously detect variations in forty inflammatory factors for exploring the pathogenesis of HAPC and the mechanisms of acclimation related to inflammatory reactions.

## 2. Materials and Methods

### 2.1. Subjects

It has been reported that men are more prone to CMS than women [18]. In this study, we only recruited men to avoid any influence of differences in sex. In all, 30 early HAPC patients (early HAPC is defined as hemoglobin concentration higher than 190 g/L in male); 24 confirmed HAPC patients (confirmed HAPC is defined as hemoglobin concentration higher than 210 g/L in male); 36 healthy control subjects who had been living on the Qinghai-Tibetan plateau (elevation: 3700-5000 meters) for at least one year, most one to two years, who were born in low-altitude and previously lived in low-altitude areas (elevation < 1000 meters); and 30 control subjects from low-altitude areas were recruited from the young male Han population between May 2010 and Oct 2011. All subjects gave informed consent. All HAPC patients in this study were diagnosed at No. 954 Hospital of the People's Liberation Army. The inclusion criterion was a diagnosis of HAPC (defined as a hemoglobin (Hb) concentration of at least 210 g/L in men). Hb and hematocrit were detected by automated hematology analyzer (Mindray BC-3000, Xiamen, China). Individuals with other diseases having similar clinical manifestations were excluded, such as acute or subacute mountain sickness, chronic respiratory diseases, primary cardiocerebral vascular disease, chronic obstructive pulmonary disease, asthma, shunt conditions, and valvular heart disease. According to our protocol, we used computer-generated randomization to select 10 subjects from each group for sample collection. All participants recruited in this portion of the study provided signed informed consent. This study was approved by the ethical committee of Xinqiao Hospital and carried out in accordance with the Declaration of Helsinki.

### 2.2. Sample Collection

Blood samples were collected after the second physical examination and after obtaining the approval of the relevant ethics committees and the informed consents of the donors. Samples were placed in clean and dry vacuum tubes without any additives. Serum was obtained after blood centrifugation at 2000 rpm for 10 minutes at 4°C. All samples were aliquoted and immediately stored at -80°C. Samples were thawed before analysis with the antibody array. One sample from the PUC group may have degraded and was not used for analysis.

### 2.3. Antibody Array Performance

The levels of inflammatory factors in the serum of the patients and the control subjects were measured using a semiquantitative human inflammatory factor antibody array assay (RayBio C-series Human Inflammation Antibody Array 3 Kit, RayBiotech, Norcross GA, USA) that detects 40 inflammatory factors (Supplementary Table S1) in one test. This assay was carried out according to the manufacturer's instructions. Briefly, the membranes were blocked with a blocking buffer at room temperature for 60 minutes and then incubated with serum samples at room temperature for 2 hours. After being washed three times with wash buffer I and three times with wash buffer II at room temperature for 5 minutes per wash, the membranes were incubated with a biotin-conjugated antibody mix for another 2 hours. The membranes were then washed again and developed for a further 2 hours with horseradish peroxidase-conjugated streptavidin at room temperature. Finally, the membranes were incubated with detection buffers for 2 minutes. The signal intensities of the individual spots on the membranes were then detected using a chemiluminescence imaging system (ImageQuant LAS4000 Scanner, USA, GE Healthcare Company). The positive, negative, and blank controls were used in all antibody microarray assays for data analysis. The relative protein levels were obtained by subtracting the background staining and normalizing the staining intensities to the positive controls on the same membrane using the RayBiotech analysis tool, which is based on Microsoft Excel software and specifically designed to analyze the data obtain with the C-series Human Inflammation Antibody Array 3 Kit.

### 2.4. Detection of Cytokines by ELISA

IL-16 and MCP-1 in the serum were detected using ELISA kits (RayBiotech, GA) according to the instruction.

## 3. Statistical Analysis

The relative protein concentrations of the inflammatory factors were statistically analyzed with IBM Statistical Package for Social Science Statistics version 20 software. Data were determined by Student's *t*-test. All values are presented as the mean ± SEM. Statistical significance was defined as a two-sided *p* value < 0.05.

## 4. Results

### 4.1. Patients' Characteristics

The characteristics of the participants in the four groups are listed in Table 1. There was a statistical difference in age between the PUC and cHAPC groups (*p* < 0.05). However, according to well-known facts, erythrocytosis is associated with hypoxia. There is no evidence indicating that age is associated with erythrocytosis until now, so age is unlikely to have an influence on the results of this study. Hb and hematocrit (HCT) levels were much higher in the HAPC groups compared to those of the two control groups (*p* < 0.05). Based on the HAPC Qinghai Diagnostic Criteria, the Chinese plateau disease diagnostic criteria, and the range of Hb levels, people from the plateau with Hb level between 150 g/L and 190 g/L were defined as normal, those with Hb level between 190 g/L and 210 g/L were diagnosed with eHAPC, and those with Hb level ≥ 210 g/L were determined to have cHAPC [19, 20]. The group classifications used in this study followed the criteria above.

### 4.2. Analysis of Serum Biomarkers in HAPC

**Figure 1:**
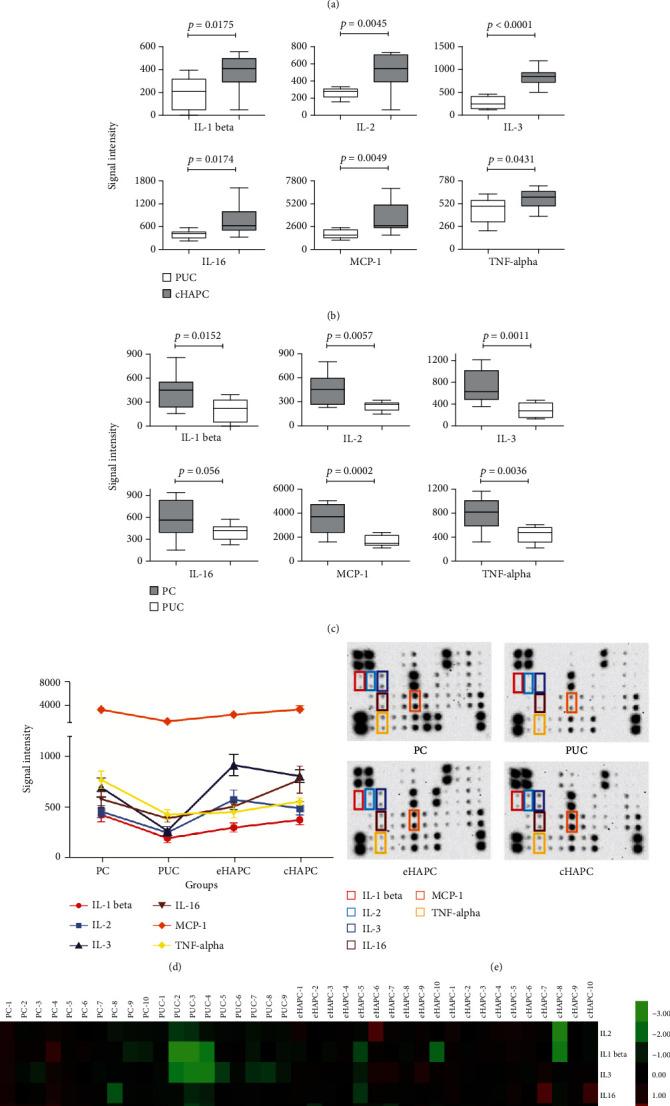
Differential expression patterns of inflammation factors by antibody array. (a, b) Array signals were scanned with an ImageQuant LAS4000 scanner, and signal values were statistically analyzed by Student's *t*-test. Differentially expressed inflammatory factors are shown by boxplots for comparisons with p values less than 0.05. The center line in the boxplots indicates the median for each data set. (c) Boxplot display of inflammation factors differentially expressed between PC and PUC. (d) Line chart of the six inflammatory factors differentially expressed among the four groups. Each line represents the level change of one of the six inflammation factors among the PC, PUC, eHAPC, and cHAPC groups. (e) The array profiles of the six inflammatory factors differentially expressed among the four groups. Each factor was measured in duplicate. Colored boxes indicate the locations of the six significantly different proteins on the arrays, and different colored boxes represent different cytokines. (f) The array data of these six inflammatory factors from the four groups are shown as a heat map, which was analyzed by Cluster 3.0 software. The levels of these proteins are represented by different colors. Low concentrations are in green, median concentrations are in black, and high concentrations are in red.

Serum samples from the four groups were incubated with the membrane arrays and the levels of 40 inflammatory factors were assessed. After comparing the expression levels of each factors from different groups, we picked out the cytokines which had significant differences between the PUC and the eHAPC (cHAPC) groups. As shown in Figure 1(a), compared with those of the PUC group, the serum levels of IL-2, IL-3, and MCP-1 in the eHAPC group were significantly increased. In addition, the expression levels of IL-1 beta, IL-2, IL-3, TNF-alpha, MCP-1, and IL-16 were all significantly increased in the cHAPC group compared with those in the PUC group (Figure 1(b), detailed data are presented in Supplementary Table S2). Currently, Hb is considered the ‘gold-standard' biomarker for the prediction and diagnosis of HAPC. The inflammatory factors mentioned above may be considerable as auxiliary diagnostic biomarkers for eHAPC and cHAPC in combination with Hb.

### 4.3. Differential Expression Patterns of Inflammation Factors among Four Groups

**Figure 2:**
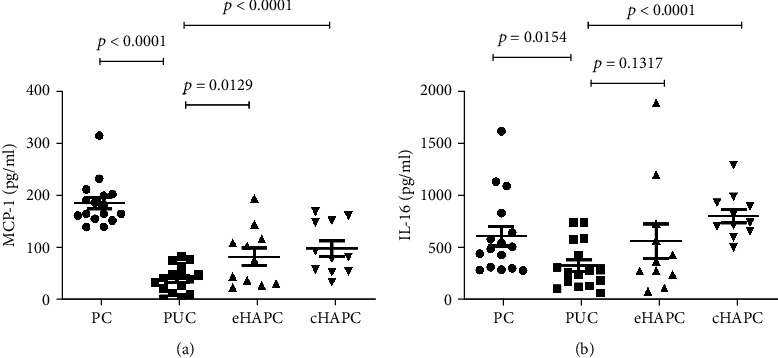
Differential expression profiles of IL-16 and MCP-1 in four groups by ELISA. The levels of IL-16 and MCP-1 in the serum from HAPC patients and control were detected by ELISA. Values were statistically analyzed by Student' *t*-test.

To explore the mechanism of high-altitude acclimation in some populations, the PC group was used in this study. Interestingly, the expression levels of IL-1 beta, IL-2, IL-3, IL-16, MCP-1, and TNF-alpha in the PUC group showed a remarkable lower value than those in the PC group (Figure 1(c)). The lower value level of these cytokines in the PUC group might be associated with high-altitude acclimation. The line chart of different cytokines in all four groups was shown in Figure 1(d). The signal intensity of MCP-1 is much higher than the values of other cytokines. There might be two reasons for this. First, the level of MCP-1 is higher than the levels of the other cytokines in every individual. Second, according to the protein array methodology, different antibody probes have different sensitivities. The probe of MCP-1 may be more sensitive than other probes, causing more signal intensity. In general, the levels of these six proteins were highest in the PC group. They were significantly lower in the PUC group and were progressively higher in the eHAPC and cHAPC groups. Among the changes of six cytokines, some (IL-1 beta, IL-2, IL-3, and TNF-alpha) are consistent with previously published studies [21–24]; however, some (IL-16, MCP-1) are novel without any report involved in HAPC. We then measured the expression levels of IL-16 and MCP-1 in other samples by enzyme-linked immunosorbent assay (ELISA). As shown in Figure 2, similar results were obtained compared with the antibody array. These results suggest that the concentration of the inflammatory factor is lower in the individuals who were already acclimatized in high altitude. However, the concentration of the factors was higher in the HAPC group, indicating that they may play an important role in high-altitude polycythemia.

### 4.4. Profiles of Inflammatory Factors from Four Groups

Arrays that are the most representative of the profiles of the four groups are shown in Figure 1(e). On the arrays, each protein was measured in duplicate using the corresponding antibodies, and the differentially expressed inflammatory factors are indicated with colored boxes. The signal intensity of the proteins on the array was proportional to their expression levels. Figure 1(e) clearly shows that these cytokines were differentially expressed in sera of the different groups. After detection, those factors with significant variations in the expression were subjected to a heat map analysis to compare the groups using Cluster 3.0 software and their expression levels are indicated by different colors (Figure 1(f)).

## 5. Discussion

To the best of our knowledge, our team was the first research group to use a high-throughput solid-protein array approach with sufficient clinical specificity and sensitivity to identify novel serum inflammatory biomarkers for HAPC. This protein array yielded six proinflammatory factors that were differentially expressed between the HAPC and PUC groups: IL-2, IL-3, and MCP-1 between the eHAPC and PUC groups and IL-1 beta, IL-2, IL-3, TNF-alpha, MCP-1, and IL-16 between the cHAPC and PUC groups.

These cytokines are sensitive markers of systemic inflammation. IL-1 beta is produced by activated macrophage and stimulates the maturation and proliferation of B cells and the release of more inflammatory mediators, which results in inflammation and tissue damage [25]. Also, high-altitude and hypoxia conditions could enhance IL-1 beta secretion [22, 25, 26]. IL-2, which is known as a T cell survival factor, plays a key role in immunity. It is involved in the proliferation, differentiation, and activation of T cells and natural killer cells and induces innate and adaptive immune responses [27, 28]. The IL-2 level was reported to be associated with acute mountain sickness [21]. IL-3 could induce the differentiation of hematopoietic stem cells into myeloid progenitor cells [29]. It is also essential for early hematopoiesis, especially in erythrocytosis [30–32], might play a role in a new mechanism in the development of HAPC [23]. TNF-alpha, a major mediator of inflammation, is involved in the pathophysiological processes of tissue damage, inflammation, and shock [24], and it has also been reported to be involved in CMS [33]. MCP-1 (monocyte chemoattractant protein-1), also been called CCL2 (chemokine (C-C motif) ligand 2), is an important proinflammatory chemokine in the CC family and could be stimulated by macrophages, fibroblast cells, B cells, and vascular endothelial cells [34]. MCP-1 could recruit monocytes, dendritic cells, and basophils to the site of inflammation and amplify inflammatory responses [34]. It has been reported that MCP-1 can be induced under hypoxic conditions in a rat model [35]. However, MCP-1 has not been reported in CMS so far. We found increased level of MCP-1 in the HAPC group compared with the PUC control in our antibody array study. The data was also validated by ELISA approach (Figure 2(a)). Therefore, MCP-1 could be a novel biomarker for HAPC. IL-16 was original described as a chemoattractant to attract activated T cells and eosinophils and was previously called lymphocyte chemoattractant factor (LCF). Afterward, it was been found to attract and activate many other cell types which expressing CD4, such as monocytes and dendritic cells [36]. It is involved in the immune response and inflammatory processes [37]. However, IL-16 has not been reported in chronic mountain sickness before. We also validate the level changes of IL-16 in different groups by the ELISA method (Figure 2(b)). Therefore, IL-16 could also be a new biomarker for HAPC.

At high altitudes, the generation of reactive oxygen species (ROS) and reactive nitrogen species (RNS) is enhanced, resulting in oxidative stress. Exposure to high altitudes can develop oxidative stress within a month [38, 39]. Oxidative stress is characterized by a biochemical imbalance between free radicals (FR) or reactive oxygen intermediates (ROI) and antioxidants [40]. The increase in ROS stimulates the activation of mediator signaling molecules, such as the transcription factor nuclear factor kappa-B (NF-*κ*B) [41], which upregulate the production of inflammatory cytokines, such as IL-1 beta or TNF-alpha [42]. Moreover, inflammatory cells secrete a large number of cytokines and chemokines that are responsible for the production of new ROI and RNS, which damage cellular lipids and result in lipid peroxidation products and lipid-derived aldehydes. These can cause numerous oxidative-stress-induced inflammatory diseases [43]. Therefore, we hypothesized that the increases in IL-2, IL-3, and MCP-1 levels may induce the initiation of HAPC via oxidative stress. After this, increases in additional inflammatory cytokines, such as IL-1 beta, IL-16, and TNF-alpha, promote the further development of HAPC via positive feedback between oxidative stress and these inflammatory factors. These proinflammatory factors may be candidate targets for HAPC therapy.

More importantly, we also aimed to reveal the mechanism by which individuals acclimate to hypoxic environments. Excitingly and interestingly, we observed a significant lower value in IL-1 beta, IL-2, IL-3, TNF-alpha, MCP-1, and IL-16 levels in the PUC group compared with that in the PC group, suggesting that these proteins are associated with acclimations to high altitude. The results showed that the downregulation of these proinflammatory factors could help individuals from the plains acclimate to high altitudes. As described above, inflammatory cytokines can activate oxidative stress and give rise to HAPC. We therefore hypothesized that organisms might suppress oxidative stress through lower the serum levels of these six proinflammatory factors as a mechanism for avoiding HAPC and facilitating the acclimation to high altitudes. In addition, the suppression of oxidative stress would reduce the production of inflammatory cytokines, which would be advantageous for the prevention of HAPC. In the present study, there were no significant differences between the PC group and the eHAPC or cHAPC groups in the levels of these proinflammatory factors, suggesting that when migrating to the plateau from low-altitude areas, some people can evade HAPC by downregulating the expression of proinflammatory factors and that other people, without this ability, will develop HAPC. Inflammation is driven by many intracellular signal pathways, such as the NF-*κ*B, MAPK, JAK-STAT, and TLR signaling pathways [44–47]. These signaling pathways might be regulated under certain factors when people acclimate to high altitudes. The mechanism by which these inflammatory factors are regulated and their importance in acclimating to high altitudes needs further elucidation.

In summary, we identified six inflammatory factors, IL-1 beta, IL-2, IL-3, TNF-alpha, MCP-1 and IL-16 that were closely related to HAPC. More importantly, these cytokines were found to be related in high-altitude acclimation. This is a novel and meaningful discovery that provides important information and new ideas for research on the pathogenesis of HAPC and high-altitude acclimation. However, our study had some limitations. The sample size used for the antibody arrays was limited, and there might be some sampling bias. Larger sample sizes and further experiments are needed to be done in the future.

## Figures and Tables

**Table 1:** Clinical data of patients and controls.

	PC	PUC	eHAPC	cHAPC
n	10	9	10	10
Age (mean ± SD, year)	29.8 ± 4.9	27.2 ± 3.5	30.2 ± 8.3	37.4 ± 9.7
Gender (male, %)	100	100	100	100
Hb (mean ± SD, g/L)	139.2 ± 2.9	160.7 ± 8.1	200.8 ± 5.4	228.1 ± 15.1
HCT (mean ± SD, g/L)	42.9 ± 1.1	47.6 ± 2.9	54.7 ± 4.5	64.9 ± 8.4

## Data Availability

The data used to support the findings of this study are available from the corresponding author upon request (corresponding author Peiyan Kong, email: peiyankong@aliyun.com, ORCID: 0000-0003-3662-3990).

## Supplementary Materials

Supplementary Materials Supplementary Table S1: map of Human Inflammation Antibody Array C3. The levels of inflammatory factors in the serum of the subjects were measured by Human Inflammation Antibody Array C3. Each of the inflammatory factor and control are shown in the map. Supplementary Table S2: the expression value of the inflammatory factor in each group. The expression level of IL-1 beta, IL-2, IL-3, MCP-1, IL-16, and TNF-alpha in each individual in each group was detected by Human Inflammation Antibody Array C3. The details were shown in the table.
