# Supplementary material for: Serum Inflammatory Factor Profiles in the Pathogenesis of High-Altitude Polycythemia and Mechanisms of Acclimation to High Altitudes
Source: Mediators Inflamm. 2021 Aug 25;2021:8844438. doi: 10.1155/2021/8844438 (PMC8413029; doi:10.1155/2021/8844438)
Supplement: Supplementary Materials — Supplementary Table S1: map of Human Inflammation Antibody Array C3. The levels of inflammatory factors in the serum of the subjects were measured by Human Inflammation Antibody Array C3. Each of the inflammatory factor and control are shown in the map. Supplementary Table S2: the expression value of the inflammatory factor in each group. The expression level of IL-1 beta, IL-2, IL-3, MCP-1, IL-16, and TNF-alpha in each individual in each group was detected by Human Inflammation Antibody Array C3. The details were shown in the table. [file 8844438.f1.doc]

**Supplementary Table S1. Map of Human Inflammation Antibody Array C3**

**
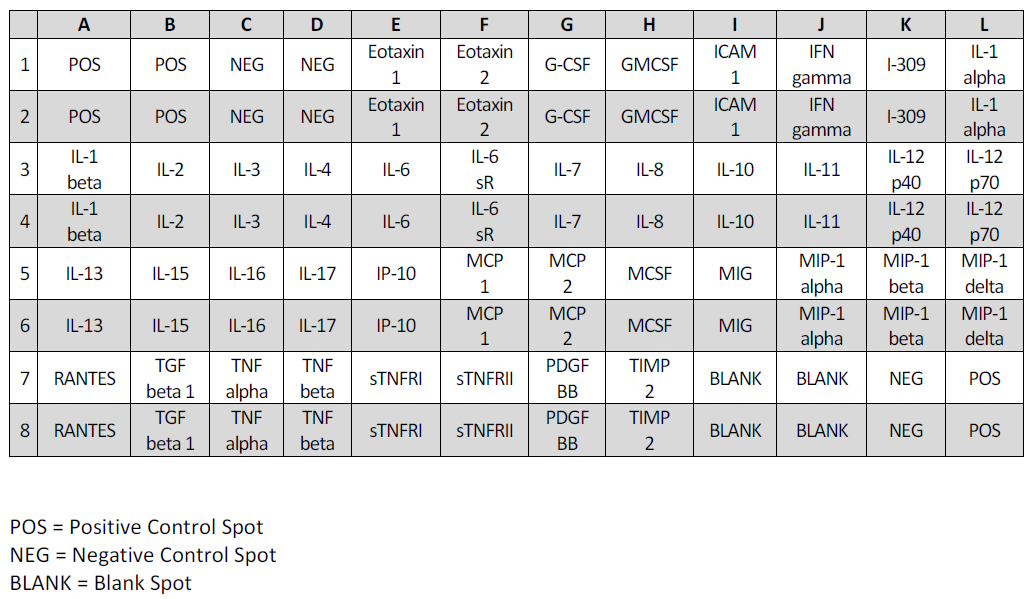
**

**Supplementary Table S2. The expression value of inflammatory factor in each group.**

|  | PC-1 | PC-2 | PC-3 | PC-4 | PC-5 | PC-6 | PC-7 | PC-8 | PC-9 | PC-10 |  | |
| --- | --- | --- | --- | --- | --- | --- | --- | --- | --- | --- | --- | --- |
| IL-1beta | 640 | 477 | 468 | 862 | 441 | 516 | 305 | 271 | 160 | 170 |  | |
| IL-2 | 810 | 331 | 530 | 670 | 578 | 434 | 486 | 242 | 266 | 283 |  | |
| IL-3 | 1223 | 468 | 340 | 1129 | 969 | 658 | 463 | 523 | 600 | 637 |  | |
| IL-16 | 933 | 500 | 440 | 946 | 797 | 628 | 676 | 146 | 382 | 406 |  | |
| MCP-1 | 4780 | 3834 | 2425 | 4145 | 3871 | 5088 | 4681 | 1609 | 2471 | 2622 |  | |
| TNF-alpha | 1095 | 964 | 903 | 1181 | 787 | 805 | 502 | 316 | 583 | 618 |  | |
|  |  |  |  |  |  |  |  |  |  |  |  | |
|  | PUC-1 | PUC-2 | PUC-3 | PUC-4 | PUC-5 | PUC-6 | PUC-7 | PUC-8 | PUC-9 |  |  | |
| IL-1beta | 278 | 29 | 1 | 72 | 294 | 395 | 189 | 344 | 212 |  |  | |
| IL-2 | 286 | 151 | 163 | 249 | 302 | 327 | 247 | 287 | 281 |  |  | |
| IL-3 | 459 | 151 | 115 | 113 | 237 | 461 | 275 | 258 | 375 |  |  | |
| IL-16 | 358 | 352 | 221 | 251 | 569 | 451 | 420 | 462 | 469 |  |  | |
| MCP-1 | 1422 | 1201 | 1282 | 1345 | 1422 | 2291 | 1911 | 1034 | 2288 |  |  | |
| TNF-alpha | 460 | 365 | 192 | 240 | 612 | 490 | 474 | 505 | 562 |  |  | |
|  |  |  |  |  |  |  |  |  |  |  |  | |
|  | eHAPC-1 | eHAPC-2 | eHAPC-3 | eHAPC-4 | eHAPC-5 | eHAPC-6 | eHAPC-7 | eHAPC-8 | eHAPC-9 | eHAPC-10 | | |
| IL-1beta | 220 | 355 | 230 | 458 | 113 | 479 | 397 | 249 | 462 | 89 |  | |
| IL-2 | 749 | 408 | 470 | 516 | 287 | 1339 | 615 | 351 | 630 | 436 |  | |
| IL-3 | 850 | 843 | 633 | 735 | 334 | 1303 | 1304 | 913 | 1381 | 921 |  | |
| IL-16 | 643 | 451 | 456 | 527 | 190 | 737 | 675 | 443 | 549 | 433 |  | |
| MCP-1 | 4179 | 3665 | 2324 | 4694 | 2040 | 1607 | 1042 | 2636 | 2448 | 2439 |  | |
| TNF-alpha | 522 | 238 | 583 | 567 | 328 | 331 | 248 | 528 | 852 | 382 |  | |
|  |  |  |  |  |  |  |  |  |  |  |  | |
|  | cHAPC-1 | cHAPC-2 | cHAPC-3 | cHAPC-4 | cHAPC-5 | cHAPC-6 | cHAPC-7 | cHAPC-8 | cHAPC-9 | cHAPC-10 | |  |
| IL-1beta | 480 | 306 | 488 | 451 | 564 | 533 | 355 | 61 | 336 | 235 |  | |
| IL-2 | 712 | 404 | 518 | 693 | 720 | 624 | 527 | 56 | 412 | 289 |  | |
| IL-3 | 1020 | 812 | 732 | 758 | 860 | 509 | 1193 | 561 | 813 | 858 |  | |
| IL-16 | 806 | 602 | 553 | 418 | 839 | 544 | 1629 | 339 | 638 | 1408 |  | |
| MCP-1 | 6425 | 4292 | 2382 | 1620 | 2227 | 2731 | 4427 | 6840 | 2526 | 2562 |  | |
| TNF-alpha | 594 | 410 | 663 | 524 | 705 | 634 | 633 | 354 | 496 | 597 |  | |
